# Changes in anti-Müllerian hormone values for ovarian reserve after minimally invasive benign ovarian cystectomy: comparison of the Da Vinci robotic systems (Xi and SP) and the laparoscopic system

**DOI:** 10.1038/s41598-024-59935-2

**Published:** 2024-04-20

**Authors:** Yunjeong Park, Ayoung Song, Junghyun Jee, Nayoung Bae, Sumin Oh, Jung-Ho Shin, Yong Jin Kim

**Affiliations:** Department of Obstetrics and Gynecology, Korea University College of Medicine, 148 Gurodong-ro, Guro-Gu, Seoul, 08308 South Korea

**Keywords:** Anti-Müllerian hormone, Ovarian reserve, Robotic surgical procedures, Laparoscopy, Benign ovarian cyst, Medical research, Reproductive disorders

## Abstract

To investigate the impact on the ovarian reserve after minimally invasive ovarian cystectomy using two platforms, the Da Vinci robotic system (Xi and SP) and the laparoscopic system. Patients underwent laparoscopic or Da Vinci robotic (Xi or SP) ovarian cystectomy for benign ovarian cysts between January 1, 2018, and December 31, 2022 at Guro Hospital, Korea University Medical center. We measured the change of AMH values (%) = [(postAMH − preAMH)] × 100/preAMH. No significant differences in preoperative age, cyst size, estimated blood loss during surgery, hemoglobin drop, length of hospital stay, adhesion detachment rate and cyst rupture rate were observed. However, the operative time was significantly shorter in the laparoscopic group than that in the robotic group (67.78 ± 30.58 min vs. 105.17 ± 38.87 min, p < 0.001) The mean preAMH and postAMH were significantly higher with the Da Vinci robotic group than with the laparoscopic group (preAMH: 5.89 ± 4.81 ng/mL vs. 4.01 ± 3.59 ng/mL, p = 0.02, postAMH: 4.36 ± 3.31 ng/mL vs. 3.08 ± 2.60 ng/mL, p = 0.02). However, the mean ΔAMH was not significantly different between two groups. ΔAMH also did not demonstrate significant differences among the three groups; laparoscopic, Xi and SP robotic. Even in the patient groups with preAMH < 2 and diagnosed with endometriosis, the ΔAMH did not show significant differences between the laparoscopic and robotic groups. The Da Vinci robotic system is no inferior to conventional laparoscopic systems in preserving ovarian function.

## Introduction

Preservation of ovarian function during surgery takes precedence as the foremost consideration for fertility preservation in the context of minimally invasive surgical procedures. Benign ovarian cysts may require surgical treatment because of torsion, pain, infertility, and decreased ovarian reserve, in which laparoscopic ovarian cystectomy has been the gold standard^1^. However, as laparoscopic surgery has been demonstrated to reduce ovarian function, determining the most suitable technique is important^2^.

The development of minimally invasive surgical methods, such as laparoscopic and robotic systems, has led to increased patient satisfaction not only in terms of pain relief but also in cosmetic aspects, including the attainment of smaller scars. In 2000, the Da Vinci robotic system was approved by the Food and Drug Administration (FDA) and began to be used in the field of surgery. The Da Vinci SP system was developed and approved by the FDA in 2018, and can operate an articulating camera and up to three robotic instruments through an umbilical incision approximately 25 mm in size^3^.

Several indicators have been used to evaluate ovarian reserve. Antral follicle count and ovarian volume are not recommended because of the variability in the menstrual cycle and lack of sensitivity^4^. Follicle stimulation hormone (FSH) has the disadvantages of significant variation and low reproducibility depending on the menstrual cycle. Estradiol is less influenced by the menstrual cycle compared to FSH, although its predictive power is limited. Inhibin B is also unsuitable as it fluctuates according to gonadotropin-realizing hormone agonist and FSH levels.

Serum anti-Müllerian hormone (AMH) is a glycoprotein belonging to the transforming growth factor-β superfamily. AMH is synthesized from the granulosa cells of the pre-antral and antral follicles. It mainly inhibits the early stages of follicular development and affects tissue growth, differentiation, and regression of fetal Müllerian ducts. Moreover, it is less affected by gonadotropin or the menstrual cycle. Therefore, AMH is currently the most widely used marker for evaluating ovarian reserve^4–6^.

This study aimed to investigate the impact of minimally invasive ovarian cystectomy using the Da Vinci robotic system (Xi and SP) and a laparoscopic system on ovarian reserve.

## Materials and methods

### Study population

This study included patients who underwent laparoscopic or Da Vinci robotic (Xi or SP) ovarian cystectomy for benign ovarian cysts between January 1, 2018, and December 31, 2022, at a single institution. All patients in this study received information about laparoscopic and robotic surgery, fully understood them, and decided on their preferred choice. This retrospective study was conducted through an electronic medical record review. This study was approved by the Institutional Review Board and Ethics Committee of the Guro Hospital, Korea University Medical Center (IRB no. 2023GR0186).

The inclusion criteria were as follows: (1) patients with confirmed AMH values within 1 month preoperatively and within 1 month–1 year postoperatively; (2) with histopathologically confirmed benign ovarian cysts; (3) women aged 15–46 years; and (4) with regular menstrual cycles (21–35 days) at the time of surgery.

The exclusion criteria were as follows: (1) pregnancy; (2) BMI ≥ 35 kg/m^2^; (3) use of medications such as oral contraceptive pills or other hormonal agents within 6 months of surgery; (4) underwent oophorectomy; (5) prior surgery for borderline or malignant tumors of the ovary; (6) history of uncontrolled infections, diabetes, hypertension, ischemic heart disease, myocardial infarction within 6 months, or serious health conditions such as liver or kidney disease; (7) undergoing cancer treatment or diagnosed with cancer within the past 5 years; (8) use of anticancer drugs, immunosuppressive drugs, or steroid drugs; (9) presence of autoimmune diseases; and (10) history of organ transplantation.

### Outcome measures

The primary outcomes were serum AMH levels including, preoperative AMH (preAMH) value, postoperative AMH (postAMH) value, and AMH change value (ΔAMH). The preAMH level was determined within 4 weeks before surgery, and the postAMH level was determined from 1 month to 1 year after surgery. The ΔAMH is expressed as a percentage value; ΔAMH = (postAMH − preAMH) × 100/preAMH

The secondary outcomes were operative outcomes, including histologic findings, operative time (min), estimated blood loss (mL), hemoglobin level change (g/dL), adhesiolysis, cyst rupture during surgery, transfusion, conversion to laparotomy, and length of hospital stay. The operative time was calculated as the time from skin incision to skin closure, including the docking time when the robotic surgery was performed. The change in hemoglobin level was calculated as the difference between the preoperative level and the level on postoperative day 1. Adhesiolysis was selected only when specific adhesion detachment was reported in the surgical records.

### Statistical analysis

All statistical analyses were performed using SPSS version 26.0 (SPSS, Armonk, NY, USA). The mean ± standard deviation or median interquartile range (IQR) was used to describe the distribution of the data after the Kolmogorov–Smirnov normality test. Differences among the three groups were evaluated using the Kruskal–Wallis test or analysis of variance for continuous variables, and multiple comparisons were performed by post hoc test using the least significant difference method. Significance was set at p < 0.05.

## Results

### Study population characteristics

In total, 132 patients were enrolled in this study. Among them, 74 underwent laparoscopic surgery and 58 underwent robotic surgery (21 with Xi robotic surgery, and 37 with SP robotic surgery).

### Comparison of operative outcomes between the laparoscopic system and robotic systems

No significant differences in age, BMI, parity, histopathologic type, position, and maximum size (cm) of the ovarian cysts were observed between the groups. The estimated blood loss during surgery, hemoglobin drop, length of hospital stay, adhesion detachment rate, and cyst rupture rate also indicated no significant differences. The operative time was significantly shorter in the laparoscopic group than in the robotic group (68.51 ± 30.99 min vs. 105.17 ± 38.87 min, p < 0.001) (Tables 1 and 2)

**Table 1:** Baseline characteristics of the patients.

	Laparoscope (N = 74)	Robot (N = 58)	*p*
Age (years)	29.96 ± 6.74	28.16 ± 6.12	0.115
BMI (kg/m^2^)	23.07 ± 4.78	22.72 ± 3.61	0.638
Parity			
Nullipara	65 (87.84%)	53 (91.38%)	0.270
Para 1 or more	9 (12.16%)	5 (8.62%)	
Histologic finding			
Endometrioma	31 (41.89%)	28^a^ (48.28%)	0.468
Mature cystic teratoma	30 (40.54%)	23 (39.66%)	0.919
Cystadenoma	9 (12.16%)	6 (10.34%)	0.746
Other cyst	4 (5.41%)	1 (0.17%)	0.246
Cyst size, in maximum (cm)	6.45 ± 2.57	7.00 ± 2.77	0.314
Cyst position			
Unilateral	56 (75.68%)	41 (70.69%)	0.523
Bilateral	18 (24.32%)	17 (29.31%)	
CA 125 (U/mL)	54.47 ± 75.19	37.43 ± 37.56	0.186

^a^Case in which a mature teratoma was identified concomitantly with an endometrioma.

**Table 2:** Operative outcomes.

	Laparoscope (N = 74)	Robot (N = 58)	*p*
Operative time (min)	67.78 ± 30.58	105.17 ± 38.87	< 0.001
Estimated blood loss (mL)	62.84 ± 93.29	93.97 ± 103.91	0.073
Hb drop (g/dL)	1.69 ± 1.00	1.87 ± 0.91	0.278
Adhesiolysis	48.65% (36/74)	36.84% (21/57)	0.177
Cyst rupture	91.67% (66/72)	85.96% (49/57)	0.318
Complications	2^a^ (transfusion)	0	
Conversion	0	0	
Length of hospital day	4.18 ± 0.73	4.29 ± 0.84	0.391

^a^Transfusion.

The mean preAMH levels were significantly higher with the Da Vinci robotic group than with the laparoscopic group (5.89 ± 4.81 ng/mL vs. 4.01 ± 3.59 ng/mL, p = 0.02). The mean postAMH was also higher with the Da Vinci robotic group than with the laparoscopic group (4.36 ± 3.31 ng/mL vs. 3.08 ± 2.60 ng/mL, p = 0.02). However, the mean ΔAMH was not significantly different between the two groups (−13.21% ± 57.10% in the laparoscopic system vs. − 18.36% ± 39.64% in the robotic system, p = 0.56) (Table 3).

**Table 3:** Serum anti-Müllerian hormone (AMH) levels (ng/mL).

	Laparoscope (N = 74)	Robot (N = 58)	*p*
Preoperative	4.01 ± 3.59	5.89 ± 4.81	0.015
Postoperative	3.08 ± 2.60	4.36 ± 3.31	0.015
ΔAMH (%)	− 13.21 ± 57.10	− 18.36 ± 39.64	0.560

AMH (%) = (postoperative AMH − preoperative AMH) × 100/preoperative AMH.

### Comparison of operative outcomes between the laparoscopic system and Xi and SP robotic systems

The patients who underwent SP robotic surgery were younger than those who underwent laparoscopic surgery (26.84 ± 6.10 years old vs 29.96 ± 6.74 years old, p = 0.034). No significant differences in BMI and parity of patients were observed among the groups. The histopathological type, position, and maximal size (cm) of the ovarian cysts demonstrated no significant differences among the three groups. Estimated blood loss (mL), hemoglobin drop (g/dL), adhesiolysis, cyst rupture, and length of hospital stay also indicated no significant differences among the groups. The operative time for Xi and SP robotic surgeries were longer than that for the laparoscopic surgery (101.62 ± 48.93 min, 107.19 ± 32.41 min vs. 67.78 min, p < 0.001) (Tables 4 and 5).

**Table 4:** Baseline characteristics of the patients.

	Laparoscope (N = 74)	Xi robot (N = 21)	SP robot (N = 37)	*p*
Age (years)	29.96 ± 6.74	30.48 ± 5.56	26.84 ± 6.10	0.034
BMI (kg/m^2^)	23.07 ± 4.78	23.23 ± 4.07	22.44 ± 3.35	0.720
Parity				
Nullipara	65 (87.84%)	19 (90.48%)	34 (91.89%)	0.529
Para 1 or more	9 (12.16%)	2 (9.52%)	3 (8.11%)	
Histopathologic finding				
Endometrioma	31 (41.89%)	8 (38.10%)	20^a^ (54.05%)	0.389
Mature cystic teratoma	30 (40.54%)	11 (52.38%)	12 (32.43%)	0.333
Cystadenoma	9 (12.16%)	2 (9.52%)	4 (10.81%)	0.939
Other cyst	4 (5.41%)	0 (0%)	1 (2.7%)	0.484
Cyst size, in maximum (cm)	6.45 ± 2.57	7.35 ± 3.54	6.80 ± 2.25	0.376
Cyst position				
Unilateral	56 (75.68%)	16 (76.19%)	25 (67.57%)	0.635
Bilateral	18 (24.32%)	5 (23.81%)	12 (32.43%)	
CA 125 (U/mL)	54.47 ± 75.19	37.94 ± 46.36	37.13 ± 32.46	0.419

^a^Case in which a mature teratoma was identified concomitantly with an endometrioma.

**Table 5:** Operative outcomes.

	Laparoscope (N = 74)	Xi robot (N = 21)	SP robot (N = 37)	*p*
Operative time (min)	67.78 ± 30.58	101.62 ± 48.93	107.19 ± 32.41	< 0.001
Estimated blood loss (mL)	62.84 ± 93.29	111.90 ± 125.40	83.78 + 79.80	0.116
Hb drop (g/dL)	1.69 ± 1.00	2.18 ± 1.27	1.70 ± 0.59	0.108
Adhesiolysis	48.65% (36/74)	33.33% (7/21)	38.89% (14/36)	0.375
Cyst rupture	91.67% (66/72)	80.95% (17/21)	88.89% (32/36)	0.386
Complications	2^a^ (transfusion)	0	0	
Conversion	0	0	0	
Length of hospital day	4.18 ± 0.73	4.43 ± 1.21	4.22 ± 0.53	0.422

^a^Transfusion.

Significantly higher preAMH levels were noted in the SP robotic surgery group than in the laparoscopic surgery group (6.35 ± 5.26 vs. 4.01 ± 3.59, p = 0.023). The postAMH value was also higher in the SP robotic surgery group than that in the laparoscopic surgery group (4.66 ± 3.54 vs. 3.08 ± 2.60, p = 0.029). However, the ΔAMH did not demonstrate significant differences among the three groups (−13.21 ± 57.10% in laparoscopic system vs. −14.63 ± 47.80% in Xi system vs. −20.47 ± 34.73% in the SP system, p = 0.772) (Table 6).

**Table 6:** Serum anti-Müllerian hormone (AMH) levels (ng/mL).

	Laparoscope (N = 74)	Xi robot (N = 21)	SP robot (N = 37)	*p*
Preoperative	4.01 ± 3.59	5.09 ± 3.91	6.35 ± 5.26	0.023
Postoperative	3.08 ± 2.60	3.81 ± 2.88	4.66 ± 3.54	0.029
ΔAMH (%)	− 13.21 ± 57.10	− 14.63 ± 47.80	− 20.47 ± 34.73	0.772

AMH (%) = (postoperative AMH − preoperative AMH) × 100/preoperative AMH.

### Comparison of ΔAMH between the laparoscopic system and robotic systems in the patients with preoperative AMH values of < 2.0

Even in the patient group with a preAMH level of < 2.0, the preAMH and postAMH values were not significantly different between the two groups. The ΔAMH was − 7.55 (IQR −48.79, 19.87) in the laparoscopic group (N = 21) and – 29.73 (IQR −59.89, 9.46) in the robotic group (N = 11), indicating no significant difference between the two groups (p = 0.72) (Table 7).

**Table 7:** Serum anti-Müllerian hormone (AMH) levels (ng/mL).

	Laparoscope (N = 21)	Robot (N = 11)	*p*
Preoperative	0.95 (0.46,1.48)	1.34 (0.74,1.87)	0.337
Postoperative	0.90 (0.33,1.35)	0.81 (0.52,1.04)	0.540
ΔAMH (%)	− 7.55 (− 48.79,19.84)	− 29.73 (− 59.89,9.46)	0.715

AMH (%) = (postoperative AMH − preoperative AMH) × 100/preoperative AMH.

### Comparison of operative outcomes between laparoscopic system and robotic system in patients diagnosed with endometriosis

In patients diagnosed with endometriosis from postoperative histopathology, preAMH, postAMH, and ΔAMH were compared by dividing the group that underwent laparoscopic surgery (N = 31) and the group that underwent robotic surgery (N = 28). The preAMH, postAMH and ΔAMH values were not significantly different between the two groups (Table 8). There was no significant difference between the pre AMH, post AMH, and ΔAMH in the laparoscopic (N = 31) and SP robot groups (N = 20) (Table 9).

**Table 8:** Serum anti-Müllerian hormone (AMH) levels (ng/mL).

	Laparoscope (N = 31)	Robot (N = 28)	*p*
Preoperative	3.90 ± 3.16	4.47 ± 3.44	0.506
Postoperative	2.49 ± 2.35	2.79 ± 2.35	0.609
ΔAMH (%)	− 23.59 ± 72.69	− 31.43±36.39	0.609

AMH (%) = (postoperative AMH − preoperative AMH) × 100/preoperative AMH.

**Table 9:** Serum anti-Müllerian hormone (AMH) levels (ng/mL).

	Laparoscope (N = 31)	SP robot (N = 20)	*p*
Preoperative	3.90 ± 3.16	4.60 ± 3.60	0.467
Postoperative	2.49 ± 2.35	2.92 ± 2.03	0.506
ΔAMH (%)	− 23.59 ± 72.69	− 28.40 ± 36.83	0.786

AMH (%) = (postoperative AMH − preoperative AMH) × 100/preoperative AMH.

### Comparison of ΔAMH between the laparoscopic system and robotic system in the patients with bilateral ovarian cysts

This study compared the rate of change in AMH level between the two platforms when bilateral ovarian cystectomy was performed using the laparoscopic and the SP robotic system. The preoperative AMH level in the laparoscopic group was 2.57 ± 2.73 ng/mL, which was significantly lower than 8.29 ± 6.40 ng/mL in the SP robotic system (p = 0.011). The postoperative AMH level in the laparoscopic group was also significantly lower than in the SP robotic system (1.90 ± 1.99 ng/mL vs. 4.97 ± 4.07 ng/mL, p = 0.029). However, the ΔAMH decreased to − 13.90 ± 53.35% in the laparoscopic group and − 39.15 ± 25.91% in the SP robotic system, the difference between the two groups was not statistically significant (p = 0.140) (Table 10)

**Table 10:** Serum anti-Müllerian hormone (AMH) levels (ng/mL).

	Laparoscope (N = 18)	SP robot (N = 12)	*p*
Preoperative	2.57 ± 2.73	8.29 ± 6.40	0.011
Postoperative	1.90 ± 1.99	4.97 ± 4.07	0.029
ΔAMH (%)	− 13.90 ± 53.35	− 39.15 ± 25.91	0.140

## Discussion

This study investigated the effects of minimally invasive surgical techniques such as, laparoscopic and robotic systems on the ovarian reserve in benign ovarian cyst surgery. The changes in AMH values were calculated as a relative value (percentage) to assess the ovarian reserve. When the changes in the AMH values were compared for each surgical platform, no significant differences were observed between the laparoscopic and robotic systems. Even in the patient group with preAMH < 2.0, in the group diagnosed with endometriosis, and in the patient group who underwent bilateral ovarian cystectomy ΔAMH did not show significant differences between the laparoscopic and robotic groups.

A systematic review and meta-analysis of minimally invasive surgery for endometriosis in 2020 revealed that robotic surgery had a longer surgical time but no inferior compared to laparoscopic surgery for length of hospitalization, intra/post-operative complication, blood loss, and conversion rate^7^. Robotic surgery can be expected to be a more sophisticated operation due to the three-dimensional view and the natural movement of robotic instruments^8^. Another study in 2020 revealed that robotic surgery in bilateral ovarian endometrioma showed a better recovery rate of serum AMH and was beneficial for ovarian function protection^9^.

In the subgroup analysis, based on an AMH value of 2, it was classified as a group < 2. We set this cut-off value by referring to the results of previous studies that the median AMH was 1.9 ng/mL among Japanese nulliparous women with a rapid decrease in fertility and serum AMH levels > 2 ng/mL, which demonstrated the highest probability of live birth^10–12^.

A committee opinion published in 2015 by the American College of Obstetricians and Gynecologists recommends evaluating ovarian function in women undergoing ovarian surgery^13^. The most widely used indicator for assessing ovarian function is AMH. AMH is an indicator of the size of the primordial oocyte pool, and it starts to increase in young adolescent women and reaches its peak at 25 years of age. Afterwards, it decreases at a rate of 0.2 ng/mL/year until age 35, and then at a rate of 0.1 ng/mL/year between ages 35 and 40. From the age of 40 onwards the median and average decrease in AMH is 0.1 ng/mL/year^10^. Over time, this decline leads to a decrease in AMH levels of approximately 5.6% per year, eventually reaching undetectable levels at menopause^6,10,14,15^.

However, the mechanism by which AMH levels decrease after ovarian surgery remains unclear. Normal ovarian tissue can fall off during the process of stripping the cyst capsule during ovarian cyst surgery and damage the functional cortex during the electrocauterization process for hemostasis. Therefore, a decrease in the number of pre-antral and small antral follicles may also reduce the AMH levels^16,17^. It is widely known that when bilateral ovarian cysts are removed, the AMH level decreases significantly compared to when unilateral ovarian cyst is removed^18–21^. This is thought to be because more damage may be caused to normal ovarian tissue during the process of removing both ovarian cysts^19^. Also the endometrioma itself may cause damage to the surrounding ovarian tissue, with decreasing serum AMH level^22^. Reduced ovarian reserve postoperatively is reported to recover at approximately 3–6 months^23,24^. Recovery of ovarian reserve could be attributed to the reperfusion of ovarian tissue, activation and rearrangement of ovarian follicles^2,25^.

The Da Vinci SP robotic system has been widely used in gynecologic surgery since its introduction, with its FDA approval in 2018. To date, no study has analyzed the surgical outcomes of ovarian cysts according to the SP surgical platform. To the best of our knowledge, this is the first study to compare surgical outcomes, particularly ovarian function, between conventional platforms and the SP robotic system.

The obese population is increasing worldwide, and this is a major burden on global health care. It is clear that the obese population is the most challenging group in surgery. The thick abdominal wall and excessive visceral fat make intra-abdominal access difficult and limit the operative field^26^. Fortunately, these problems have been solved due to the development of minimally invasive surgery and improved operator skills. In particular, robotic systems are known to be more useful in the obese group due to their short learning curve, 3D visualization, freer movement, and tremor cancellation^27^. However, it was difficult to compare the obese group in this study. The patients with BMI ≥ 35.0 kg/m^2^ were excluded from the analysis. This is because in Korea, 4.3% of women have a BMI of 30.0–34.9 kg/m^2^, and 0.75% have a BMI ≥ 35.0 kg/m^2^, which is a big difference from the US group of 39.8% with a BMI ≥ 30.0 kg/m^2^^28,29^.

The Xi and SP robotic surgeries required longer operative time than that of the laparoscopic surgery (101.62 ± 48.93 min, 107.19 from 32.41 min vs. 67.78 min, p < 0.001), and was calculated from skin incision to closure time. This could be calculated by considering the docking and undocking times; however, owing to the limitations of the retrospective study, determining exactly how many docking and undocking times the robot performed during each surgery was not possible. Moreover, the Da Vinci SP robotic system was introduced to our institution in 2020, and further research is needed to evaluate its proficiency and effectiveness, given its recent implementation in early stage surgeries.

This study had some limitations. First, the study is retrospective in nature. The evaluation of the AMH value was not performed in a batch period, depending on the operator; therefore, the measurement of the postAMH value was widely done within one year. Owing to the nature of the tertiary institution, many patients were sent back to the 1st or 2nd institution postoperatively; therefore, only few patients had their AMH measured multiple times. Second, the sample size was small as the robotic group was further divided according to the two systems, SP and Xi.

Compared to the existing laparoscopic system, the robotic system does not demonstrate a significant difference in the preservation of the ovarian reserve; therefore, it will be widely selected as an option for minimally invasive surgery.

## Conclusion

The Da Vinci robotic system is no inferior to conventional laparoscopic systems in preserving ovarian function.

## Data Availability

The data underlying this article will be shared on reasonable request to the corresponding author.
